# Remarks on the Gravity of Any Discharge from the Ear

**Published:** 1894-06-23

**Authors:** Wheelton Hind


### Medical Progress and Hospital Clinics.

[*The Editor will be glad to receive offers of co-operation and contributions from members of the profession. All letters should be addressed to* The Editor, The Lodge, Porchester Square, London, W.]

### REMARKS ON THE GRAVITY OF ANY DISCHARGE FROM THE EAR.

By Wheelton Hind, M.D.B.S., F.R.C.S.

It should be laid down as an axiom in surgery that a discharge from the ear should never be allowed to go untreated, for not only is such a condition a very satisfactory trouble to treat in the early stages, but if allowed to go on untreated may be the starting point of the most disastrous mischief.

The most potent causes of ear trouble are the exanthemata, of which measles and scarlet fever are answerable for the majority of cases, and after these chronic throat troubles, such as granular pharyngitis adenoids, and enlarged tonsils.

In these diseases suppuration of the middle ear is due to an extension of the mischief from the throat along the eustachian tube, and owing to the absence of a free escape for inflammatory discharges tension is raised, and perforation of the membrana tympanum takes place, with the free discharge of pus from the external ear, pain then ceases, and the popular idea that a little discharge from the ear is rather a healthy thing than otherwise is often supported by the apathy of the medical attendant, and the case is allowed to run on and become chronic. There are several reasons why a cure so rarely results spontaneously. The middle ear is a bony cavity, and has therefore no contractile or expulsive powers and pus therefore accumulates, and the visible discharge is only an overflow; moreover owing to the changes in direction and concavo-convexity of the floor of external auditory canal some of the overflow is allowed to remain within it, keeping the epithelial lining in a sodden state, if it does not infect it septically. Such being the morbid anatomy, it is plain that all discharges from the ear should be made the subject of treatment, and this is best carried out by frequent careful syringing with astringent antiseptic lotions. I myself make use of a wash containing corrosive sublimate 1 in 1,000, and alum sulph. gr. x ad. ℥j. After syringing, the passages must be carefully dried with absorbent wool on a probe, and then some powdered boracic acid should be blown in, which acts at once as an antiseptic and dessicator. It will be noticed that on syringing out the ear in early cases that a clot of puriform material is washed out nearly every time. This is the result of the damming back of discharges behind the remains of the tympanic membrane. This treatment may have to be continued for weeks, but it will eventually be found to be successful, more especially if the general health of the child be attended to by the free exhibition of iron tonics and stimulating food. Throat conditions demand attention, enlarged tonsils or adenoids should be removed. Chlorate of potash gargles or chloride of ammonia inhalations will be beneficial, and often, too, it will be well to gently politzerise the patient to open up the eustachian tubes and remove all discharges which may be lodging within them.

The treatment of cases which have become chronic is more tedious, but, nevertheless, must be persevered with on the same lines as I have indicated above. There is, however, greater need for antiseptic measures, as the discharge becomes thin and foul, while curdy flakes of puriform material become left in the middle ear and passages; the epidermis is sodden and is continually being shed, and in many cases there is also present that very troublesome affection polypus, which is not, indeed, a true polypus, but a mass of granulative tissue springing from the membrane or bone, and shaped by the external auditory meatus. This indicates often that the disease has spread to the bone, and is most difficult to eradicate, as it returns freely after removal. I have tried in vain caustic applications to the base after removal, such as silver nitrate, zinc chloride, and liquor plumbi subacetate, and only succeeded in eradicating the polypus by curretting. This I believe to be the best method of dealing with such cases, for if left sooner or later more mischief will ensue through necrosis and suppuration. An anæsthetic will be often necessary in doing this small operation, and if necessary—that is if the bones of the middle ear themselves be found necrotic—there need be no hesitation in removing them, as it is probable that the condition of hearing will be in no way any further impaired by so doing. Careful dressing of the middle ear must be enjoined for some time after the operation, and the greatest care must be taken to keep it dry.

The sequela of neglected ear troubles are both numerous and dangerous, calling out all the resources of the surgical art. The commonest trouble met with is mastoid abscess, or an extension backwards into the mastoid cells of the inflammatory process, which become filled with pus. The general history given in these cases is that the patient has suffered from a running at the ear for some time, which has lately ceased. Pain and headache has ensued, and there are often signs of inflammation present. If a careful examination of the external auditory canal be made, signs of curdy pus and often a septic odour will be perceptible. If the pus has made its way through the mastoid, as it often does, the ear will stand out from the head and there will be an angry swelling behind it. If the case has not advanced as far as this there will be pain and tenderness over the mastoid when touched, some œdema of the skin, often deafness. At the same time there will be signs of fever, the temperature often running up to 103.

In this condition no time should be lost in expectant treatment, but the pus must be evacuated by cutting down upon the mastoid, which must then be perforated either by a trephine drill or gouge. The latter is the instrument I use myself, and I gradually bore my way into the cells until I reach pus. It must be borne in mind that in the very young the mastoid cells are very little developed. After reaching the pus and washing it out the walls of the cavity should be explored with a probe. It may at times be found that the inner wall has become absorbed, and that the dura mater separating the lateral sinus can be felt. Means should then be taken to re-establish the communication between the mastoid and the middle ear, so that lotion can be syringed through from one aperture to the other, but in making this connection care must be taken to avoid the facial nerve, which is otherwise very liable to be damaged. Having established a free passage and syringed out well with antiseptic lotion, it is well to blow a powder composed of iodoform and boracic acid into the cells and external meatus, and to plug the wound with iodoform gauze. Very violent hæmorrhage may be met with during this operation from the wounding of the mastoid vein, which is often of large size, but this is very easily controlled by pressure.

The after-treatment consists of carefully syringing out the wound and ear, and keeping the parts antiseptic. As a rule cases do very well and are soon able to get about again, in my own experience relapses being very rare.

The other sequelæ of chronic ear mischief are more dangerous, but space will only allow me to briefly allude to them. Abscess of the brain may occur in two situations after ear disease, either in the tempero-sphenoidal lobe or the cerebellum, but it is very difficult to diagnose in any case which of these localitiss is the seat of the mischief. The chief symptoms are optic neuritis and delayed cerebration, patient is drowsy and comatose, but can be roused. The only course to pursue in these cases is to trephine, and if pus be not found in the one situation to seek it in the other.

More dangerous and indeed absolutely hopeless is general suppurative meningitis, which is occasionally met with after ear disease. I once trephined in a case of this sort hoping to find an abscess in the brain, the symptoms calling for interference being convulsions and rigors. There seemed to be some slight improvement for a few days, but the convulsions returned and terminated fatally. The post-mortem showed general suppurative meningitis secondary to middle ear mischief. I had at the time of operation opened the mastoid cells and evacuated pus.

The only other sequela of chronic ear disease, that I shall mention, is septic infection of the lateral sinus. This can be treated, and many cases have been reported of brilliant success. The internal jugular is tied in the neck and the lateral sinus trephined, the septic clot removed, and the cavity syringed out and packed with antiseptic gauze.

Prevention is, however, better than cure, and therefore it is a mistake to permit any discharge from the ear to continue. Insurance companies are becoming alive to the risk, and many require an answer to the question, "Has there ever been a discharge from the ear?" so that it is both of vital and pecuniary importance that early treatment should be adopted and persevered with.

